# Routine laboratory parameters to support decision on parenteral nutrition in palliative care

**DOI:** 10.3389/fnut.2023.1173106

**Published:** 2023-11-03

**Authors:** Lea Kum, Elisabeth L. Zeilinger, Dagmar Vohla, Anna Kitta, Nadine Brunevskaya, Feroniki Adamidis, Franziska Ecker, Eva K. Masel, Brigitte Mayr-Pirker, Alexa L. Meyer, Bärbel Sturtzel, Gudrun Kreye, Matthias Unseld

**Affiliations:** ^1^Division of Palliative Medicine, Department of Medicine I, Medical University of Vienna, Vienna, Austria; ^2^Department of Clinical and Health Psychology, Faculty of Psychology, University of Vienna, Vienna, Austria; ^3^Academy for Ageing Research, Haus der Barmherzigkeit, Vienna, Austria; ^4^Department of Geriatric Medicine, Christian Doppler University Hospital, Paracelsus Medical University, Salzburg, Austria; ^5^Division of Palliative Care, Department of Internal Medicine II, University Hospital Krems, Karl Landsteiner University of Health Sciences, Krems, Austria

**Keywords:** decision tree, parenteral nutrition, routine laboratory parameters, prognostic score, biomarkers, palliative care, cancer, cachexia

## Abstract

**Introduction:**

Parenteral nutrition (PN) is widely used in palliative care (PC), but there is limited evidence to support its use at the end of life (EOL). This aim of this was to investigate the relationship between routine laboratory parameters and survival in patients receiving PN, and to develop a decision tree model to support clinicians decide whether to start or forgo PN.

**Methods:**

The laboratory parameters of 113 patients with advanced diseases who were admitted to a specialized palliative care unit (PCU) were analyzed at two points in time: T0 = before PN, T1 = two weeks after initiation of PN. Univariate Mann-Whitney U-tests and multivariate linear regression models, as well as a decision tree analysis were computed; all in relation to survival time.

**Results:**

The final regression model was significant with *p* = 0.001 (adjusted R2 = 0.15) and included two predictors for survival time after PN initiation: the CRP/albumin ratio and urea at T1 (ps = 0.019). Decision tree analysis revealed three important predictors for classification of survival time after PN initiation: CRP, urea, and LDH (all at T0).

**Discussion:**

The decision tree model may help to identify patients likely to benefit from PN, thus supporting the clinical decision whether or not to start PN.

## 1. Introduction

In the palliative medical field, parenteral nutrition (PN) is a controversially discussed topic. Especially when it comes to end of life (EOL) care there is little evidence on the benefit of PN and termination of PN was described to be one of the ethically most challenging decisions for health care professionals (1). The latest European Society of Medical Oncology (ESMO) guidelines on cancer cachexia in adult patients suggest that the closer to the end of life patients are, the less invasive nutrition should be (2). The guidelines recommend that PN should not be administered when the expected prognosis is less than three to six months (2, 3). The definitions for EOL vary. Depending on the literature, the EOL time span may refer to the last year or six months or even days and hours of life (4, 5). Hereinafter EOL will concern the last six months of life.

PN is a medical intervention with risk factors that need to be understood and considered to ensure beneficial use. Therefore the three principals [1] indication, [2] therapeutic goal, and [3] patient consent need to be fulfilled before the initiation of PN (6). Next to pleurocentesis and ascites drainage, PN is a commonly used medical application at the EOL (7, 8) which requires an initially invasive procedure for administration. A structured framework was suggested to decide the necessity of an invasive intervention for a patient receiving palliative care (PC), also including PN (9). In particular for decision making at the EOL there are several approaches to define the requirements. All of them have in common that quality of life (QoL), the indication or benefit of the intervention and the patient’s will should be taken into account (6, 9). A very strong indication for continuance of PN is to satisfy hunger. If there is no clear medical indication for ongoing PN treatment, this must be discussed with the patient or the legal representative. The use of PN without a clear therapy goal may be considered futile medical care and should be stopped. At this point, the patient needs to be carefully informed of clinician’s decision, as nutrition can be an emotionally charged subject (6, 9). Prognosis can also be an important factor to consider when it comes to deciding whether to start or forgo PN. On the one hand, communication and empathic skills are essential for delivering the decision to the patients and their families (10), which can be challenging for both medical and nursing staff (11). On the other hand, more data is needed to support the decision in terms of indication, benefit, and prognosis in regard to PN.

In PC, especially at the EOL, the indication for PN might also differ from other medical fields. Patients who present with weight loss and loss of appetite, but are still able to partially eat orally, are often started on PN treatment at the end of life (12). Negative effects of this invasive procedure must be considered. The risk of infections (13–15) next to a minimal chance of improvement of the nutritional status should be considered. Also, a lack of improvement in QoL has been observed as well as no gain in overall survival (OS) (3,16). Furthermore, an increase in inflammatory activity in patients under PN was even associated with decreased OS. Therefore, several biochemical markers such as albumin, liver function parameters, or C-reactive protein (CRP) have been described to evaluate their prognostic potential. With regard to inflammatory activity, higher levels of CRP were shown to be significantly associated with a negative outcome in terms of OS in different patients receiving PN (17). However, there is a lack of predictive markers that might help in the decision of suspending PN in the palliative setting (17–19).

As prognosis is a commonly used marker in decision-making, the ambition of predicting survival with an objective score is of significant value. One existing score is the objective palliative prognostic score (OPPS) for patients with advanced cancer. It includes heart rate > 120/min, white blood cells >11,000/mm^3^, platelets <130,000/mm^3^, serum creatinine level > 1.3 mg/dL, serum potassium level > 5 mg/dL, and no history of chemotherapy. By using this score Chen et al. could predict in an accurate way that a patient would die in 7 days (20). However, this was not specific for patients receiving PN.

Other scores such as the Palliative Prognostic Index (21), the Palliative Prognostic (PaP) Score (22) and the Prognosis in Palliative Care Study Score (PiPS) (23) often rely on subjective variables. These include patients’ symptoms or condition and physicians’ experience. However, routine laboratory blood parameters are commonly available for each patient and are objective diagnostic tools in the daily decision making process of physicians (24, 25). Therefore, an objective prognostic model including routine laboratory parameters might help to aid in the decision whether to start or forgo PN in PC patients at the EOL. However, for PC patients, such a prediction model has not been established, yet.

The main objectives of our study were [1] to investigate the relationship between routine laboratory parameters and patient survival under PN and [2] to build a decision tree model based on routine laboratory parameters to support decision-making related to the initiation of PN. The predictive model is intended to help clinicians make the difficult decision of whether or not to start PN. Having an objective score to contribute to this fundamental care decision may improve person-centered PC and EOL.

## 2. Materials and methods

### 2.1. Study design

In this retrospective data analysis, the laboratory parameters of patients admitted to the Division of Palliative Medicine of the Medical University of Vienna between January 2016 and January 2019 have been analyzed.

### 2.2. PN regimens

The decision regarding whether to administer PN was made by the dietician of the PCU in consultation with the medical staff according to the individual needs of each patient. The PN administered was NuTRIflex® Omega special (625 mL bag with 740 calories, 35 g of proteins, 90 g of carbohydrates and 25 g of fat; B. Braun Melsungen AG, Germany, 2014), with added supplements of Soluvit (vitamins: b1, b6, b12, c, nicotinamide, pantothenic acid, biotin and folic acid; Fresenius Kabi Austria GmbH, Austria, 2013), Vitalipid (contains vitamins: a, d2, e and k1; Baxter Deutschland GmbH, Germany, 2015) and Trace (contains trace elements: Fluorine (F), Iodine (I), Molybdenum (Mo), Iron (Fe), Copper (Cu), Manganese (Mn), Selenium (Se) and Zinc (Zn) as well as electrolytes; Fresenius Kabi Austria GmbH, Austria, 2018). The administration of PN usually takes place overnight. The targeted number of calories was calculated by the dietician based on the individual needs of the patients, with a mean of 1,475 kcal/d.

### 2.3. Study participants and data collection

All patients admitted to the palliative care unit (PCU) who were started on PN were included in the analysis. The final sample comprised *N* = 113 patients. We collected baseline data as age, sex and body mass index (BMI) and laboratory parameters from the electronic database of the Medical University of Vienna. After exporting data from the electronic system, we performed a random data check to assure correctness of the automated export. To identify the dynamics of the laboratory parameters under PN administration, two time points were set retrospectively: First, on the day of admission (T0), i.e., before PN initiation, and second, two weeks after the initiation of PN (T1). To ensure data protection, personalized files were only stored on password-protected computers. A pseudonymized file was used for analysis.

### 2.4. Statistical analysis

For sample description, median, interquartile range (IQR), and total range were used. Table 1 lists all predictor variables included in analysis. Rational for inclusion was based on availability of data. If available, laboratory parameters at two points in time, T0 and T1, as well as the difference between these two points in time were included, to capture changes over time. Based on a recent study (17), we also included the CRP/albumin ratio as predictor. Further aspects included were BMI and sex. Survival time after initiation of PN was specified as primary outcome. As the recommendation for initiation of PN is a survival time of at least three months (2, 3), this cut-off was applied to split the sample into two subsamples containing patients living shorter and longer than three months after initiation of PN, respectively.

**Table 1:** Characteristics and predictors for analysis in both subsamples.

	Less than three months (*n* = 93)		More than three months (*n* = 20)		*p value*	Effect size (*r*)
	Median (IQR)	Range (Min-Max)	Median (IQR)	Range (Min-Max)		
Age [*years*]	60 (52–69.5)	20–85	61.5 (54–70)	29–78	0.596	0.05
BMI [*kg/m^2^*]	20.3 (17.4–22.7)	12.5–29.3	18.7 (17.2–21.1)	14.4–28	0.266	0.11
Bili T0 [*mg/dl*]	0.4 (0.3–0.8)	0.1–11.5	0.4 (0.3–0.8)	0.1–2.9	0.699	0.04
Bili T1 [*mg/dl*]	0.4 (0.3–1)	0.1–16.9	0.4 (0.3–0.5)	0.2–5.1	0.522	0.07
Albumin T0 [*g/L*]	29.1 (25–34.2)	17.8–44	29.7 (25–34.6)	0.4–40	0.728	0.03
**Albumin T1** [*g/L*]	25.6 (21.7–30.4)	16–38.7	30.5 (26.9–32.5)	23–37	**0.014**	**0.27**
LDH T0 [*U/L*]	194.5 (154–290.8)	114–1878	205 (141–266)	70–761	0.91	0.01
LDH T1 [*U/L*]	205 (159–311)	41–630	206 (157–286.3)	106–578	0.867	0.02
GOT T0 [*U/L*]	24.5 (18–42.5)	6–332	25 (16–40)	12–332	0.746	0.03
GOT T1 [*U/L*]	28 (19–44.8)	11–324	26 (18.8–48.3)	13–151	0.86	0.02
GPT T0 [*U/L*]	18 (11–33)	5–289	14 (9.8–31.5)	5–374	0.589	0.05
GPT T1 [*U/L*]	21.5 (14.3–41)	8–251	33 (16.3–49.8)	8–139	0.371	0.10
gGT T0 [*U/L*]	88.5 (41.5–236)	10–2,884	66 (37–300)	15–706	0.879	0.01
gGT T1 [*U/L*]	158.5 (75–385)	13–2,190	155.5 (53.5–395)	21–925	0.95	0.01
AlkP T0 [*U/L*]	111.5 (78.3–211.8)	38–1,690	89 (71–309)	58–826	0.734	0.03
AlkP T1 [*U/L*]	164 (103–338)	41–2,496	164 (107.3–339)	47–920	0.8	0.03
CRP T0 [*mg/dl*]	6.3 (2.9–13.2)	0–46.4	5.1 (2.3–13.5)	1–23	0.541	0.06
CRP T1 [*mg/dl*]	8.9 (4–18.9)	0.4–41.3	7.2 (5.1–10.3)	0.5–18.5	0.218	0.13
Leukocytes T0 [*G/L*]	8.1 (6.2–13.6)	1.9–65.4	7.5 (4.6–10.2)	1.4–18.8	0.192	0.12
**Leukocytes T1** [*G/L*]	10.6 (7.2–13.8)	2–99.6	7.3 (5.3–11.4)	1.2–12.5	**0.053**	**0.22**
Sodium T0 [*mmol/L*]	136.5 (133–140)	122–149	137 (134–138)	131–145	0.903	0.01
Sodium T1 [*mmol/L*]	138 (132.5–141.5)	126–154	137 (134–139.8)	124–143	0.648	0.05
Creatinine T0 [*mg/dl*]	0.7 (0.6–1.1)	0.3–4	0.6 (0.5–0.8)	0.3–1.5	0.141	0.14
Creatinine T1 [*mg/dl*]	0.7 (0.5–1.4)	0.2–6	0.7 (0.5–0.8)	0.3–1.7	0.247	0.13
Magnesium T0 [*mmol/L*]	0.7 (0.7–0.9)	0.4–1.3	0.7 (0.7–0.8)	0.4–0.9	0.771	0.03
Calcium T0 [*mmol/L*]	2.1 (2–2.2)	1.1–3.4	2.2 (2–2.3)	1.9–2.8	0.282	0.10
Potassium T0 [*mmol/L*]	3.8 (3.4–4.1)	2.6–6	3.9 (3.2–4.1)	2.8–4.9	0.823	0.02
Potassium T1 [*mmol/L*]	4 (3.5–4.3)	0.5–5.8	4.1 (3.8–4.4)	3.2–6.4	0.301	0.11
**Urea T0** [*mg/dl*]	19 (11.7–29.3)	2–76	11 (9.3–20.5)	3.9–34.3	**0.016**	**0.23**
Uric Acid T0 [*mg/dl*]	4.9 (3.4–8.3)	1.2–26.4	4.1 (3.1–6.4)	2–9	0.193	0.13
CRP/albumin ratio T0	0.2 (0.1–0.5)	0–1.9	0.2 (0.1–0.5)	0–5.3	0.969	0.00
CRP/albumin ratio T1	0.3 (0.1–0.8)	0–1.9	0.3 (0.1–0.4)	0–0.8	0.092	0.18
Bili diff	0 (− 0.2 - 0.3)	−10.6 - 14.4	0 (− 0.2–0.1)	−1.1 - 3.3	0.499	0.08
**Albumin diff**	−4.4 (− 8.2 - 1.2)	−18.7 - 11	−0.1 (− 4.1–4.1)	−8.1 - 28.6	**0.037**	**0.24**
LDH diff	6.5 (− 28–52.8)	−285 - 245	−9.5 (− 41.5–29.8)	−85 - 176	0.383	0.10
GOT diff	3 (− 4–13.3)	−238 - 247	2.5 (− 5.8–12.3)	−181 - 28	0.57	0.06
GPT diff	3 (− 6–18)	−175 - 193	3 (− 6–23)	−235 - 37	0.882	0.02
gGT diff	54.5 (− 14.3–174.3)	−1,386 - 1341	77 (− 4–215)	−188 - 307	0.797	0.03
AlkP diff	37 (1–92)	−233 - 1085	29 (− 14–113.3)	−65 - 354	0.639	0.05
CRP diff	2.6 (− 2.4–7.4)	−30.8 - 35.2	2 (− 2.8–4.2)	−12.6 - 6.9	0.165	0.15
Leukocytes diff	1.5 (− 3.5–5.9)	−41.5 - 34.3	1.3 (− 2.2–2.9)	−10.1 - 3.7	0.434	0.09
Sodium diff	0.5 (− 3–4.8)	−11 - 15	0 (− 2–3)	−12 - 8	0.656	0.05
Creatinine diff	−0.1 (− 0.2–0.1)	−1.7 - 4.3	0 (− 0.2–0.2)	−0.4 - 0.7	0.745	0.04
Potassium diff	0.1 (− 0.3–0.6)	−2.9 - 2	0.2 (− 0.2–0.6)	−0.7 - 3.4	0.439	0.09
**CRP/albumin ratio diff**	0.1 (0–0.4)	−1.25 - 1.86	0.1 (− 0.2–0.1)	−5.1 - 0.3	**0.059**	**0.21**

Parameters with an effect size *r* > 0.2 are highlighted in bold. Diff = difference in the respective parameter from T0 to T1. BMI, body mass index. Bili, bilirubin. LDH, lactate dehydrogenase. GOT, serum glutamic oxaloacetic transaminase. GPT, serum glutamic pyruvic transaminase. gGT, gamma-glutamyl transferase. AlkP, alkaline phosphatase. CRP, C-reactive protein.

In a first step, to compare patients who lived shorter vs. longer than three months, Mann–Whitney U-tests were applied. For these initial explanatory tests, we did not rely on significance values but rather on effect sizes, and calculated the effect size r for each test. Effect sizes are more informative than value of ps, because they are independent of sample size and represent scale-free indices (26, 27). Interpretation followed Cohen’s guidelines, with r = 0.1 resembling a small effect, *r* = 0.3 a medium effect, and *r* = 0.5 a large effect (28). In a second step, predictors with an effect of *r* > 0.2 in univariate analysis were entered in a stepwise regression analysis to examine their multivariate association with survival time. Variance Inflation Factors (VIFs) were examined and indicated no multicollinearity between predictors in the regression model. Due to high skewness, survival time was log(x + 1) transformed, which has been shown to be a robust method for skewed data (29). Significance level for determining relevant indicators in regression analysis was set to 5%.

In a final step, a decision tree analysis was conducted as complementary method to establish a classification model for predicting survival time. The goal of a decision tree model is to make predictions or decisions by recursively partitioning a dataset into subsets based on available data, aiming for accurate and interpretable results. Decision trees are a popular machine learning algorithm for classification tasks. They are particularly useful because of their simplicity and interpretability (30). In decision tree analysis, patients are divided into subgroups that differ maximally from each other with respect to the outcome variable based on the values of predictor variables. The present outcome variable was survival time after initial assessment (when PE was initiated). In contrast to other analysis, the results of a decision tree model are robust even when predictors are highly intercorrelated. As growing method, CART (Classification And Regression Trees) was applied. All analysis were performed in IBM SPSS Statistics, v.27. The procedure for CART in SPSS is based on Breiman and colleagues (31).

## 3. Results

The total sample comprised *N* = 113 patients (55% female) who received PN. Mean age was 60.1 years (SD = 13.1). The most frequent diagnosis was gastrointestinal cancer, followed by cancer of the reproductive organs, ear nose throat cancer, and lung cancer (see Table 2).

**Table 2:** Diagnosis in the total sample and subsamples.

	Total sample (*N* = 113)		Less than 3 months (*n* = 93)		More than 3 months (*n* = 20)	
Tumor origin	*n*	%	*n*	%	*n*	%
Gastrointestinal	49	43.4	41	44.1	8	40
Reproductive organs	12	10.6	11	11.8	1	5
ENT	11	9.7	7	7.5	4	20
Lung	11	9.7	10	10.8	1	5
Blood	6	5.3	5	5.4	1	5
Breast	5	4.4	4	4.3	1	5
Sarcoma	4	3.5	3	3.2	1	5
NET	3	2.7	1	1.1	2	10
CUP	3	2.7	2	2.2	1	5
Brain	3	2.7	3	3.2	0	0
RCC/Urothelial	2	1.8	2	2.2	0	0
Thyroid	1	0.9	1	1.1	0	0
Mesothelioma	1	0.9	1	1.1	0	0
**Nonmalignant**						
Cystic fibrosis	1	0.9	1	1.1	0	0
Chronic kidney disease	1	0.9	1	1.1	0	0

ENT, ear nose throat tumor. NET, neuroendocrine tumor. CUP, cancer of unknown primary. RCC, renal cell carcinoma.

All patients analyzed in this study were already deceased at the time of data analysis, therefore survival time was available for the total sample. A total of *n* = 93 patients lived less than three months after initial assessment, and *n* = 20 patients lived three months or longer. The characteristics of these two samples are depicted in Table 1. Mann–Whitney U tests revealed a significant difference between these two groups in three parameters: Albumin at T1 with an effect of *r* = 0.27, urea at T0 with an effect of *r* = 0.23, and the difference in albumin from T0 to T1 with an effect of r = 0.24. However, although not statistically significant due to the small sample size, the following two parameters also showed an effect size of *r* > 0.2: Leukocytes at T1 with *r* = 0.22, and CRP/albumin difference from T0 to T1, with *r* = 0.21. Results of all univariate analysis are given in Table 1.

In a next step, a multivariate stepwise regression analysis was computed. The five parameters with *r* > 0.2 were entered as predictors (albumin at T1, urea at T0, albumin difference T0 to T1, leukocytes at T1, and CRP/albumin difference from T0 to T1) and log-transformed survival time was used as dependent variable. The final multivariate regression model was significant with *p* = 0.001 (adjusted R2 = 0.15). Results indicate that only the CRP/albumin difference and urea at T0 were significant predictors for survival time in a multivariate linear model. Results of the regression analysis are detailed in Table 3.

**Table 3:** Results of stepwise regression analysis.

	Estimate	*SE*	95% *CI*		*p*
			LL	UL	
Intercept	0.066	0.008	0.05	0.081	<0.001
CRP/albumin ratio	−0.014	0.006	−0.025	−0.002	0.019
Urea T0	−0.001	0	−0.001	0	0.019

The dependent variable, survival time after initial assessment, was log (x + 1) transformed due to high skewness. For the total model, R2 and R2 adjusted was.17 and 0.15, respectively, with *p* = 0.001.

**Figure 1:**
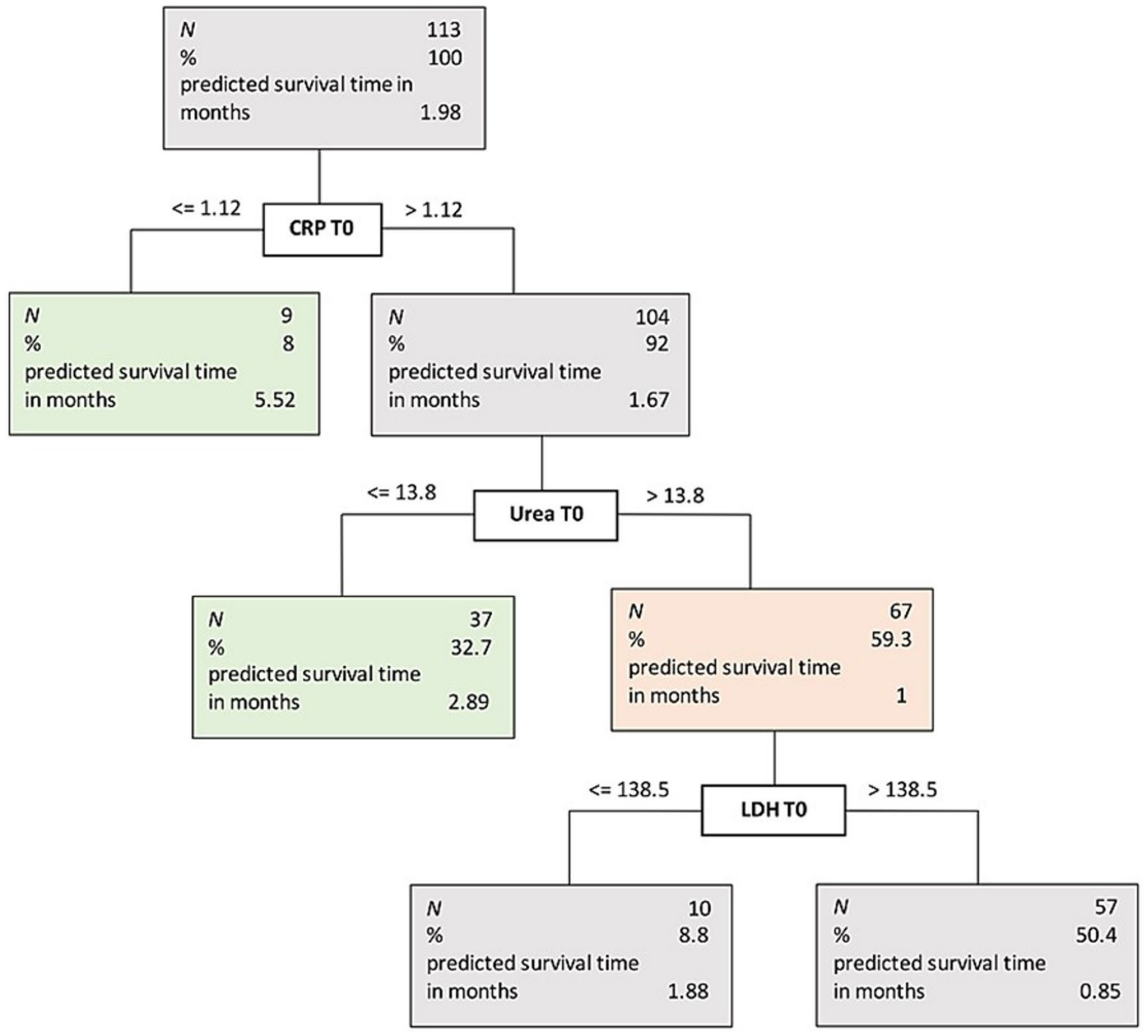
Decision tree.

In a final step, the decision tree method was applied to establish a model for supporting decision-making on whether or not to initiate PN. Results revealed three important predictors for classification of survival time after PN initiation (see Figure 1): CRP, urea, and LDH (all at T0). Patients with CRP ≤ 1.12 had a mean survival of 5.5 months. Patients with CRP > 1.12 were further split into groups by urea, with a cut-off value of 13.8. Patients below this value, had a mean survival of 2.9 months; patients above were further split according to their LDH level with a cut-off of 138.5. Patients below this cut-off had a mean survival time of 1.8 months, and patients above the cut-off had a mean survival time of 0.9 months.

Based on this model, we could establish three clinically meaningful groups of patients: The first group is characterized by a CRP level ≤ 1.12; the second group is characterized by a CRP level > 1.12 and a urea level ≤ 13.8; and the third group is characterized by CRP level > 1.12 and an urea level > 13.8. The first two groups have an estimated survival about or above three months, whereas the third group has an estimated survival time below three months.

## 4. Discussion

Considering the relatively frequent use of PN combined with nearly no evident tool that supports the clinician in the decision-making process, we consider our findings of great interest. The literature on decision tools for starting PN in patients with advanced cancer is sparse (2). The findings of this study add the insight that a combination of routine laboratory parameters, including CRP, urea and LDH, should be considered as prognostically relevant when considering the initiation of PN. Despite the fact that QoL and alleviating symptoms are the primary goals in EOL care (5) our findings can be a useful information for clinicians since the decision to initiate or stop nutritional treatment is considered one of the most challenging tasks (1). Therefore, our decision tree model might support healthcare professionals when it comes to these ethical decisions at the EOL. For clinically relevant decisions, the decision tree model and cut-offs as outlined in Figure 1 can be applied.

A Japanese study showed that beliefs and perceptions about PN and hydration were important not only for the patients but also for family members (10). Food and nutrition are of eminent importance for patients with advanced cancer because lack of sufficient nutrition is related to fear of death for many patients and their relatives. Since baseline anxiety and stress levels are usually elevated in cancer patients (32, 33) any potential additional stressor should be managed carefully. Previous studies in PC settings suggest that many patients and family members wish to receive nutritional support when patients become unable to take sufficient nourishment orally. At this time period, the negative impact of cachexia, such as anorexia, reduced food intake, muscle loss and body weight loss, become apparent (34–37). Moreover, most patients wished to receive PN and hydration, whereas many hesitated to receive enteral tube feeding under the same conditions (36). Furthermore, an unmet need for nutritional support, or PN and hydration, may be a source of eating-related distress, not only for patients but also for their family members, which needs to be alleviated by integrated palliative, supportive, and nutritional care (38).

Recent guidelines suggest to use life expectancy as decision tool, indicating that if estimated life expectancy is less than three months, PN should not be started (2, 3). In clinical practice, estimation of prognosis can be difficult. Therefore, the use of a prognostic model to estimate patients survival is of great interest for patients with advanced cancer in a PC setting (17, 20–23, 39, 40). Only a few of these models are designed especially for patients on PN for example the objective prognostic score by Llop-Talaveron and colleagues (17) that retrospectively looked at the data of 460 patients who received PN. As prognostic markers, they identified CRP, prealbumin, albumin, CRP/prealbumin and CRP/albumin. They found CRP/albumin to be statistically significant for exitus, infection, sepsis and liver failure. Based on their findings, they suggested a systematic use of the CRP/albumin score before initiating PN (17). Other studies have also shown that for patients receiving PN, an increase in CRP, as well as white blood cell count and worsening of renal function parameters, are linked to a worse outcome (18, 19).

Notably, the present methods of analysis, the regression model and the decision tree model, yielded slightly different results regarding prognostic markers. In the regression model, CRP/albumin difference and urea at T0 were significantly associated with survival time after PN initiation. In the decision tree, the clinically relevant markers for deciding whether to start PN were CRP and urea. It is common for these two analyses, which are inherently different, to yield different results. The regression model investigates a linear relationship between the prognostic markers and the dependent variable, survival time, independent of the length of survival. The decision tree aims to discriminate between two groups of patients, those who live longer than three months and those who live shorter than three months, without assuming linearity, yielding clinically meaningful results. The present study differs from former findings since our cohort solely consists of patients in a palliative setting. The comparable study from Llop-Talaveron et al. did include all inpatients who did receive PN (17). In our study, we only included patients with advanced diseases who were admitted to a PCU. Since the PCU, is a tertiary center for PC, most patients showed complex symptoms and often were admitted in a very advanced stage of their disease, explaining why OS in general was not longer than six months. In their randomized controlled trial Bouleuc et al. found a life expectancy shorter than three months to be the cut-off for initiating PN (3).

The latest ESMO guidelines from 2022 suggest not to start with PN when survival is considered less than three to six months (2). From a retrospective view, the majority of our cohort was not fit for the initiation of PN, since 93 of 113 patients died in less than three months after initial assessment. This could lead to the conclusion that clinicians were unaware that PN was not indicated at the time of initiating treatment. On the one hand this could be due to negative effects of PN on OS like infections (17, 18). On the other hand, prognosis of the patients might have been estimated to be better. It is commonly known that clinicians tend to overestimate the predicted survival time (41). Thus the need for an objective easy-to-use tool led to the development of a variety of scores such as the ‘Objective Palliative Prognostic Score’ (OPPS) (20) or the laboratory prognostic score for respiratory malignancy (R-LPS) (39). These scores were designed to predict short term survival. The OPPS predicts survival over the next seven days while the R-LPS predicts death within 14 days (20, 39). As already mentioned, for the decision whether to start or forgo PN a survival time of more than three months is of interest (2).

The R-LPS was designed by analyzing nineteen blood parameters of 649 terminally ill patients. Among other laboratory parameters, CRP was described as an independent factor for survival (39), whereas the OPPS uses the white blood cell count as an inflammatory prognostic marker (20). Our findings support CRP as a prognostic marker. In the group of patients with the longest survival (5.52 months), CRP was below 1.12 mg/dL (see Figure 1). Our findings show that prognosis of patients with advanced diseases was better when blood urea was lower. This is also supported by the R-LPS, where blood urea is described as an independent factor for 14-day survival (39). The CRP/albumin ratio was linearly related to survival time, as shown by regression analysis, but was not part of the final decision tree model. Therefore, in our analysis, CRP was the more relevant factor in deciding on PN than the CRP/albumin ratio.

Lactate dehydrogenase (LDH) was found to be a predictive factor in the ‘Objective Prognostic Score’ (OPS), a score designed to predict the three-week survival for advanced cancer inpatients in South Korea and prospectively validated (42, 43). In the present decision tree analysis, LDH was found to be a relevant marker, but was not clinically relevant for the decision to start PN treatment. As Figure 1 indicates, LDH only divided the subsample with a median survival time of one month into two groups of 1.88 months and 0.85 months, respectively. Since both groups are far below three month, LDH was not considered clinically relevant in our analysis. However, it might be an interesting predictive marker for patients with a longer mean survival time as the OPS and our findings suggest (42, 43).

One limitation of the present study is the sole use of retrospective data. Planning a prospective trial evaluating prognostic and predictive factors to screen for patients who will benefit from PN could lead to ethical difficulties. The wish for PN can be very prominent in patients with advanced cancer, even if the life expectancy is less than three months and although the wish might be futile. Another limitation is the short period of survival of patients enrolled in the analysis. Further studies need to be conducted to assess the period of survival where patients still benefit from PN treatment and also to validate our findings.

Another major limitation of the present study, related to its retrospective nature, is the lack of detailed information on the indication for PN in the patient collective. Furthermore our study lacks to assess improvement of QoL and alleviation of symptoms. There is no documentation available concerning the nutritional status, the degree of cachexia or an indication like gastrointestinal obstruction or hunger. In the palliative medical field indication for starting PN might differ since the primary goal is improvement of QoL (6, 9, 44). Therefore, PN might also be initiated in patients with no sings for malnutrition but with symptoms like hunger or functional impairment. A large retrospective cohort study that included patients with advanced cancer who died in French hospitals did investigate factors that are associated with PN treatment within the last seven days of life. They identified malnutrition to be significantly associated with the use of PN in PC patients (45).

It is also worth mentioning the lack of data to differentiate whether patients received PN only or had oral food intake alongside. The unavailability of data on how much of the prescribed PN amount was actually administered to the individual patient, can also be considered a limiting factor. In general the heterogeneity of the patient collective is mentioned as a limiting factor in earlier studies an can be applied to the current study as well (46). Due to this heterogeneity individual nutritional interventions did prove to be beneficial before (47, 48).

Furthermore, the study population includes PC patients with different tumor origins. When attempting to predict survival using only laboratory parameters, tumor origin should be considered as a confounding variable. Some comparable previous studies focused on only one tumor entity (39, 40, 49). Others had an even broader subject sample, including non-cancer patients (17). For individual decision making, it might be helpful if future studies could differentiate according to tumor origin. However, it should be noted that PC cohorts will always be heterogeneous and physicians should always focus on improving QoL as the main goal of care. Our findings, as well as previous prognostic scores (21–23) should only help in decision making.

Since this was a retrospective study also the possible PN associated complications could only be analyzed in retrospect. One of the most important complications are infection which we retrospectively identified as clinically relevant when patients were started on antibiotic treatment. This was the case for six patients during the time period of interest. Discussing futile PN with patients and their families is one of the most difficult tasks for oncologists, often more difficult than offering PN. This factor also underlines the importance of PC skills among physicians, which should ideally be taught early in professional training using teaching methods that encourages self-reflection (50). Discussing with patients that they are not feasible to receive PN because they have adverse prognostic factors and will likely not benefit from PN requires more than one sensitive and empathic EOL conversation with these patients. Guidelines for such discussions should also be included in prospective study protocols investigating prognostic and predictive factors for providing PN to patients with advanced cancer.

## 5. Conclusion

Our findings suggest that CRP, the CRP/albumin ratio and urea are the most important baseline markers for predicting survival after PN initiation. Based on the results of this study, clinical decision making could be informed by the established decision tree model, which could support the identification of patients likely to benefit from PN based on CRP and urea prior to PN initiation. These findings may help clinicians in daily practice to decide when to initiate or forgo PN treatment in terminally ill patients. If used systematically, the decision tree model developed in this study could reduce overtreatment at the end of life.

## Data availability statement

The raw data supporting the conclusions of this article will be made available by the authors, without undue reservation.

## Ethics statement

The studies involving humans were approved by Ethics Committee of the Medical University of Vienna, Austria. The studies were conducted in accordance with the local legislation and institutional requirements. The ethics committee/institutional review board waived the requirement of written informed consent for participation from the participants or the participants’ legal guardians/next of kin because it was a retrospective study on routinely collected data.

## Author contributions

LK, MU, and EZ: conceptualization. NB and EZ: methodology. EZ: software. FA, FE, AK, GK, LK, EM, BM-P, and DV: validation. FA, AK, LK, EM, MU, AM, BS, and DV: investigation. FA, FE, AK, LK, EM, MU, and DV: resources. NB, LK, EZ, and MU: data curation. GK, LK, DV, MU, and EZ: writing—original draft preparation. FA, NB, FE, AK, GK, LK, EM, BM-P, AM, BS, and DV: writing—review and editing. MU and EZ: supervision. LK and EZ: project administration. All authors contributed to the article and approved the submitted version.
